# Zika virus induces monocyte recruitment in the immunocompetent adult brain driving chronic inflammation

**DOI:** 10.3389/fimmu.2025.1597776

**Published:** 2025-07-04

**Authors:** Josefina Garcia Diaz, Soo-Jeung Park, Lou Legouez, Tina Comlekoglu, Ashley Beck, Chia-Yi Kuan, Young S. Hahn

**Affiliations:** ^1^ Beirne B. Carter Center for Immunology Research, University of Virginia, Charlottesville, VA, United States; ^2^ Department of Microbiology, Immunology and Cancer Biology, University of Virginia, Charlottesville, VA, United States; ^3^ Department of Neuroscience, Center for Brain Immunology and Glia (BIG), University of Virginia School of Medicine, Charlottesville, VA, United States

**Keywords:** zika (ZIKV), monocytes, microglia, neuroinflammation, immunocompetent adult brain

## Abstract

Zika virus (ZIKV) is a neurotropic pathogen linked to neuropathogenesis in adults, causing conditions such as Guillain-Barré syndrome (GBS) and fatal encephalitis. Intracranial injection of virus in immunocompromised mice have shown neuroinflammation and subsequent brain damage. However, the mechanisms underlying ZIKV-induced neuroinflammation in immunocompetent adult mice via peripheral infection remain unclear. To investigate this, we utilized a murine model of ZIKV infection via footpad injection. Our findings reveal that acute ZIKV infection at 4 days post-infection (4 dpi) induces significant apoptosis and neuroinflammation in the adult brain, persisting up to 28 dpi. Notably, ZIKV infection triggers apoptosis in the hippocampus and cortex—key regions involved in memory—and induces early immune cell infiltration. Additionally, microglial activation occurs following infection at 7 dpi, with viral RNA detected in the brain. Bulk RNA sequencing of the hippocampus at 28 dpi further reveals the activation of inflammatory pathways, underscoring the prolonged neuroinflammatory response in the infected brain. Microglial activation is likely driven by infiltrating monocytes, as inhibiting monocyte recruitment reduced the expression of microglial activation genes. These results suggest that targeting monocyte-induced inflammatory mediators could be potential therapeutic interventions for ZIKV.

## 1 Introduction

Zika virus (ZIKV) is a single-stranded positive RNA virus belonging to the Flaviviridae family. ZIKV is primarily transmitted through the bite of a mosquito, though sexual and vertical transmission can also occur (1, 2). Global travel and climate change have expanded the populations of human carriers and the geographical range of mosquito vectors to transmit the virus (3). Of particular concern is ZIKV, which has shown an increased infection rate from 1998-2018, with Brazil having reported 440,000-1,300,000 suspected cases in 2015. While ZIKV infection in pregnancy is well-known for causing microcephaly in the fetal brain, adults infected with ZIKV had detectable viral RNA in the brains and impaired cognitive function (4, 5). ZIKV infection in adults has been associated with severe neurological outcomes, including acute encephalitis, Guillain-Barré syndrome (an autoimmune neuropathy causing weakness, paralysis, or even death), and long-term cognitive impairments (6, 7). Around 20% of symptomatic adults report rash, fever, conjunctivitis, and headache (8). Approximately 0.3% of ZIKV-infected individuals develop neurological sequelae, with 75% of these cases resulting in GBS (9, 10). In rare but concerning cases, ZIKV has been linked to acute encephalitis and long-term cognitive decline (4, 5, 11–13). Some studies have further explored connections between viral infections and neurodegeneration; for example, ZIKV exposure *in vitro* has been shown to increase amyloid-β production, and ZIKV-infected brain organoids exhibit Alzheimer’s-like pathology, including elevated Aβ and phosphorylated tau (14–18).

Microglia, the brain’s resident macrophages, are central to clearing extracellular debris and releasing pro-inflammatory cytokines and neurotoxins (19, 20). Pathogenic microglia can exacerbate brain injury by inducing pro-inflammatory A1-astrocytes (21). Microglial-derived neurotoxins can be packaged into exosomes and released into the CNS, amplifying neuroinflammation (22), and tau-laden exosomes from microglia contribute to Alzheimer’s disease pathogenesis (23). ZIKV infection has been shown to activate microglia in both immunocompromised mouse models and intracerebroventricular (ICV) injection studies (24). Furthermore, microglia can communicate with astrocytes and prolong immune activation, disrupting the BBB (51). However, the role of microglia in immunocompetent models via peripheral infection remains understudied. Monocytes, derived from bone marrow, infiltrate the brain following infection and may exacerbate neurological damage. ZIKV-infected monocytes exhibit enhanced transmigration across the blood-brain barrier (BBB), upregulate adhesion genes, and release exosomes containing infectious viral particles (24–28). These findings underscore the importance of investigating ZIKV’s effects on monocyte-microglia interactions to better understand and mitigate virus-induced neuroinflammation and cognitive dysfunction.

To better understand ZIKV neuropathogenesis and assess potential therapies, it is crucial to establish an animal model that mirrors human disease. Intracerebral ZIKV inoculation in immunocompetent adult mice demonstrates CNS infection and damage (29). In this model, antiviral responses are driven by microglia, infiltrating monocytes, and macrophages, suggesting that innate and adaptive immunities play a key role in ZIKV-associated encephalitis. Another widely used model, Ifnar1 knockout mice in a C57BL/6 genetic background, lacks type-I IFNα/IFNβ responses and shows heightened susceptibility to viral replication and neurological damage (30). However, due to their compromised immune systems, these mice are not ideal for testing antiviral agents targeting innate immunity. To address this, a murine model was developed using wild-type C57BL/6 mice, with transient inhibition of type I IFN signaling through anti-IFNR antibody injection one day before ZIKV infection. This model is susceptible to infection and develops severe neuropathological changes, making it valuable for studying ZIKV neuropathogenesis (31, 32).

In this report, we investigated how ZIKV affects the immunocompetent adult brain by using a transiently inhibited IFNAR mouse model. We found that during acute infection, ZIKV infection induces robust expression of inflammatory genes, such as *IL-6*, *TNFα*, and IL-1β in the brain. Notably, infected mice exhibited prolonged neuroinflammation and sustained microglial activation. Inhibition of monocyte recruitment attenuated microglial activation markers, implicating infiltrating monocytes as key drivers of neuroinflammatory microglial responses. These findings suggest that ZIKV-infected monocytes contribute to CNS injury through microglial activation. The understanding of ZIKV-associated CNS damage will help for the development of targeted therapies to prevent long-term neurological damage in adults.

## 2 Materials and methods

### 2.1 Virus

The Uganda isolate (strain MR766) of zika virus was obtained from Dr. Michael Gale. For making new viral stocks, Vero cells were cultured and infected with an MOI of 0.05 of ZIKV. 10 mL of serum free media (DMEM) was added for 4 hours while shaking, followed by adding 20mL of 10% FBS-containing media. Cells were then harvested and stored in -80C after successful viral replication at 3–5 days post infection. Plaque assays were performed to quantify infectious virus titers. Serial 10-fold dilutions of thawed viral stocks were prepared in serum-free DMEM, beginning with a 1:10 dilution (50 μL virus into 450 μL medium) and proceeding to 10^-8^. Vero cell monolayers were washed with serum-free media and infected with 200 μL of each dilution in duplicates. Plates were incubated at 37°C for 2 hours, with gentle swirling every 15 minutes to promote viral infection. Following incubation, cells were washed three times with serum-free media to remove unbound virus. An overlay medium containing either 1% agarose or 0.3–0.6% Avicel in DMEM (supplemented with 10% FBS and NaHCO_3_) was applied gently (4 mL/well). Plates were incubated for 5 days. After incubation, cells were fixed in 10% formaldehyde in PBS and stained with 0.1% crystal violet. Plaques were counted in wells containing 5–100 plaques, and titers were calculated as PFU/mL using the formula: Plaques/(dilution factor * volume added)= pfu/mL. Multiple dilutions were used to determine titer accuracy, and the assay was repeated for reproducibility.

### 2.2 Real-time quantitative PCR

For RNA isolation, Trizol was used following the Trizol induced RNA isolation protocol (Invitrogen). Following RNA isolation, the RNA was resuspended in nuclease free water and the quality/concentration of the RNA was assessed by nano-drop 2000 spectrophotometer (Thermo Scientific). For brain tissue, 1500ng of RNA was used for cDNA. cDNA synthesis was done using High-capacity RNA-tocDNA kit (Applied Biosystems). Real-time PCR was performed on a StepOnePlus system (Applied Biosystems). Primers were used for target gene quantification using SYBR green master mix (Applied Biosystems). Target gene expressions were determined using comparative cycle threshold (ΔΔCT) technique and results normalized to HPRT. Please see **Table 1** for primer sequences.

**Table 1:** Primer sequences: List of primers used for RT-qPCR with the corresponding forward and reverse sequences.

Gene	Forward sequence	Reverse sequence
*HPRT*	GTGTTCTAGTCCTGTGGCCA	TCAAAAGTCTGGGGACGCAG
*NS5*	AARTACACATACCARAACAAAGTGGT	TCCRCTCCCYCTYTGGTCTTG
*PRM*	TTGGTCATGATACTGCTGATTGC	CCCTCCACGAAGTCTCTATTGC
*IL6*	GAGGATACCACTCCCAACAGACC	AAGTGCATCATCGTTGTTCATACA
TNFα	GGTGCCTATGTCTCAGCCTCTT	GCCATAGAACTGATGAGAGGGAG
*IL1β*	CTTTCGACAGTGAGGAGAATGAC	CAAGACATAGGTAGCTGCCACAG
*APOE*	CTGACAGGATGCCTAGCCG	CGCAGGTAATCCCAGAAGC
*CD86*	TGTTTCCGTGGAGACGCAAG	TTGAGCCTTTGTAAATGGGCA
*CH25H*	CTGCCTGCTGCTCTTCGACA	CCGACAGCCAGATGTTAATCA
*CX3CR1*	ACCGGTACCTTGCCATCGT	ACACCGTGCTGCACTGTCC
*P2RY12*	TTTCAGATCCGCAGTAAATCCAA	GGCTCCCAGTTTAGCATCACTA
*RIG-I*	GAGAGTCACGGGACCCACT	CGGTCTTAGCATCTCCAACG
*ISG15*	CTGAAGAAGCAGATTGCCCAGAAG	CGCTGCAGTTCTGTACCACTAGC

### 2.3 Mice, microscopy

The mouse experiments were approved by the University of Virginia Animal Care and Use Committee (ACUC). This study was conducted under approved bioethics protocols, including IBC protocol #077–99 for biological agents. Further all animal procedures were approved by the Institutional Animal Care and Use Committee (IACUC) under protocol number 2720-10-22. CX3CR1-GFP and CCR2-CreER;R26R-EGFP (Ai6) mice were kindly provided by Dr. Chia-Yi Kuan’s lab, UVA professor. Mice were injected with IFNAR via IP injection (2mg/mL, 200uL injected) one day prior to footpad injection of ZIKV (10^4/10^5/10^6, 50uL). RS-102895 from SIGMA, a CCR2 inhibitor, was administered via IP injection at 1 hpi and at 24 hpi. Mice were euthanized using 60uL of Avertin (10 g of 2,2,2-tribromoethyl alcohol with 10 ml of tert-amyl alcohol to make 100% Avertin, freshly diluted to 2.5% in saline before each use) via IP injection to avoid brain injury that other methods may impose. Decapitation was performed to ensure death as a secondary confirmation method, along with the removal of the brain tissue for studies. Following euthanizing, mouse blood was collected, and perfusion was performed using 1XPBS. Tissue was then harvested and stored in appropriate medium for downstream applications.

For microscopy, harvested brains were kept in 4% PFA overnight and then placed in 30% Sucrose, before being frozen in OCT. Brain was sliced, and slices were placed on a histological slide and stored until staining. Tissue staining was performed following a modified STAR protocols 100499, Sep 17, 2021. Primary antibodies used were SALL1 (eBioscience), and GFAP (sc33673), and secondary antibodies used were AF647 (Invitrogen). UVA microscopy core Leica Thunder widefield microscope was utilized for data acquisition. Exposure, gain and intensity were kept the same for all samples to ensure rigor. Additionally, images were captured on the same day for fair comparisons. Images were captured at various resolutions 40x and 100x. Analysis of images was performed using IMARIS 10.2.0 software version. Using IMARIS software identical region areas and analysis parameters were applied across all images to ensure consistency. Cells were counted in IMARIS using the spots feature with their representative intensities being measured through spots. This work used the Leica thunder TIRF widefield imaging microscope in the Molecular Imaging Core Facility which is supported by the University of Virginia School of Medicine, Research Resource Identifiers (RRID): SCR_025472.

### 2.4 IHC

Mouse brains were harvested and stored in 10% Formalin for 48 hours before being transferred to cassettes in 70% ETOH. Brains were submitted to UVA Research Histology Core for paraffin embedding and H&E/Oil red staining. For tissues used in H&E analysis H&E staining and paraffin slides, work was done in the Research Histology Core Facility which is supported by the University of Virginia School of Medicine, Research Resource Identifiers (RRID): SCR_025470. TUNEL assay was performed using the ab206386 TUNEL Assay kit HRP-DAB following manufactures instructions. TUNEL-positive cells were manually counted in a defined region of interest (ROI) in the hippocampus/cortex.

### 2.5 Flow cytometry

Bone marrow immune cells were isolated from mouse femurs and 1mL of RBC buffer was added to cell pellet to remove red blood cells. After centrifugation, cells were washed with 1X PBS and then resuspended in FC buffer. Brain immune cells were isolated using a digestion buffer consisting of: Papain at 0.1 mg/mL, DNase I 1ug/mL, and 1X HBSS without Ca++ and Mg++. Briefly, after brain tissue was isolated from mice, they were placed in 1X PBS to wash as the next tissues were isolated. Following the end of the harvest, brains were transferred to a small cell culture dish where they were cut in half, and one section was used for PCR/IHC, and the other to continue flow analysis. The one hemisphere for flow was minced to around small <1mm pieces and 1mL of the digestion buffer was added. The minced brains were then transferred to a 15mL tube where 6mL of digestion buffer was added. Brains were then placed in 37°C for 30 minutes with gentle agitation throughout. Following incubation, brains were filtered through a 100um filter and rinsed with sample preparation medium consisting of 1X HBSS without Ca++ and Mg++ and 10% FBS. Brains were centrifuged at 1350rpm for 10 minutes and 30% Percoll was added to the cell pellet. Centrifugation again at 700g for 10 minutes with the brake off resulted in a cell pellet. The isolated immune cells were washed with 1X PBS and then resuspended in FC buffer for flow analysis. Antibodies used for staining were eBioscience F4/80-FITC, BD pharmigen CD11B-perCP-Cy5.5, Invitrogen CD45-APC, Invitrogen LY6G-PE, eBiosceince, and Invitrogen live/dead fixable violet dead cell stain. Results from experiment were analyzed using FlowJo.

### 2.6 Bulk sequencing

Mice were harvested and the hippocampus was quickly isolated and stored in RNAlater before processing. RNA was isolated from the tissue as mentioned previously and library preparation was conducted by the UVA genome analysis and technology core. Data analysis was conducted in Rstudio using Deseq2 and GO enrichment analysis to search for gene pathways. Library preparation and sequencing was done by the University of Virginia School of Medicine Genome Analysis and Technology Core, RRID: SCR_018883.

### 2.7 Luminex analysis

Mouse BMDMs were cultured and infected with MR766 for 24hr. Supernatant was obtained and stored at -80°C until used for assay. Protein from cells were isolated using Cell Lysis Buffer II (Invitrogen) supplemented with 1mM PMSF and Protease Inhibitor Cocktail (Thermofisher), according to the manual instructions. Protein concentrations were determined through use of Pierce BCA kit (Thermofisher) and concentrations were diluted to 0.5 μg/μL using 1X PBS. Mouse 32 Plex Luminex assay was run by UVA’s Flow Cytometry Core Facility.

### 2.8 ELISA

ELISAs were performed according to Biolegends ELISA protocol for mouse IL1*β*. BMDM supernatants were used in assessing protein amounts. Briefly, ELISA plates were coated with 100uL of IL1*β* capture antibody overnight at 4C and after washing the plates assay diluent provided was used for the blocking step. Next after washing, the samples and standards were added for 2 hours at RT. Detection antibody was then added for an hour followed by Avidin-HRP for 30 minutes at RT. Lastly, TMB substrate solution incubation in the dark for 15 minutes was performed before adding the stop solution and reading the wells. Protein amounts were assessed using an absorbance reader at 450nm within 15minutes.

### 2.9 Statistics

GraphPad Prism version 9 was used for conducting statistical analysis of data obtained throughout the experiments. Unpaired t-tests were conducted and values of p < 0.05 were regarded as statistically significant. Statistical tests were performed for controls vs treated groups. Data represents the mean ± SD with ^∗^p < 0.0332, ^∗∗^p < 0.0021, and ^∗∗∗^p < 0.0002, ^∗∗∗∗^p < 0.0001.

## 3 Results

### 3.1 Acute ZIKV infection induces cell apoptosis in the hippocampus and cortex of the adult brain leading to chronic neurological deficits

**Figure 1:**
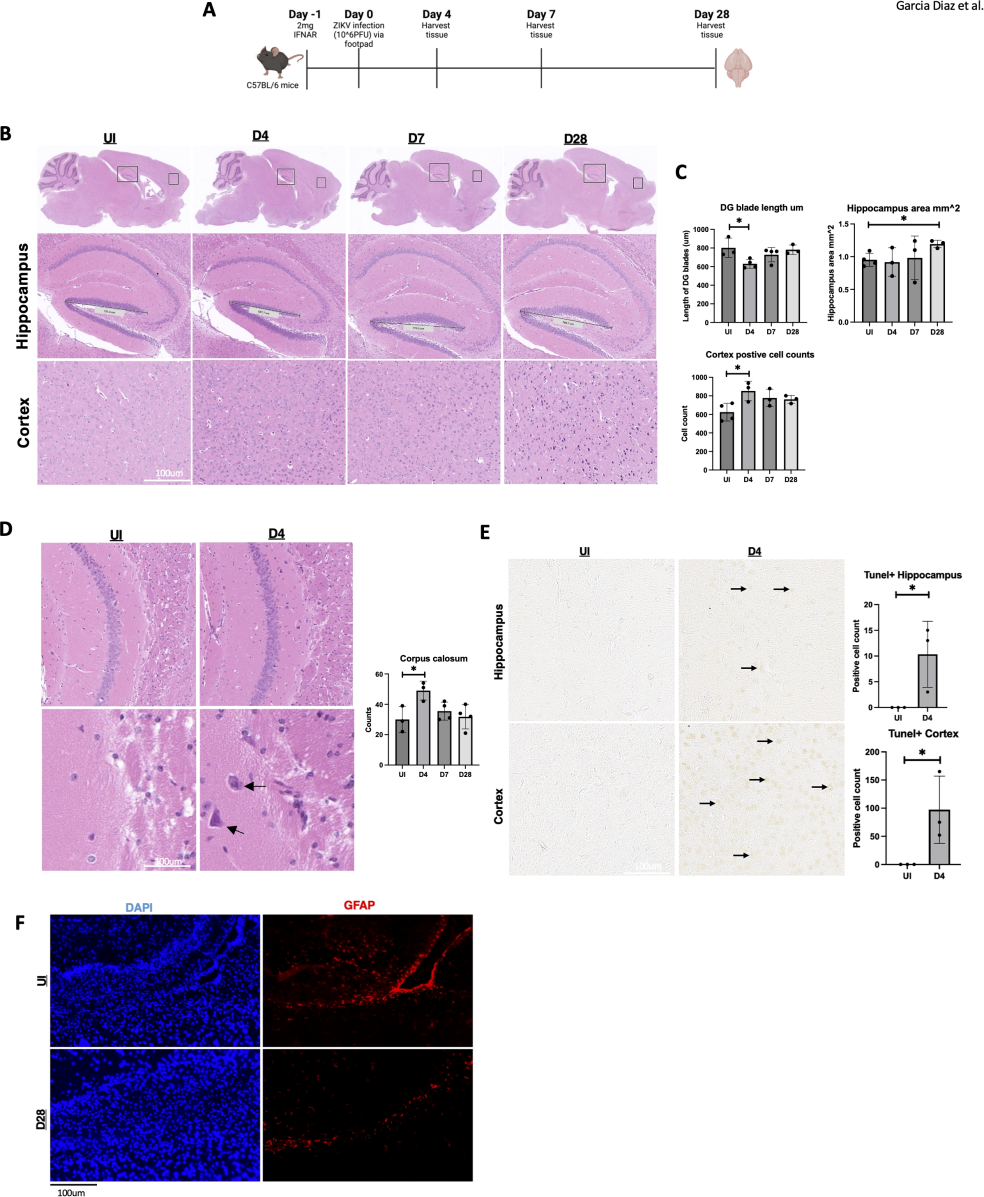
Hippocampal area altered by ZIKV followed by cell death in the adult hippocampus and cortex. **(A)** C57BL/6 mice were injected with 10^6 PFU of ZIKV via footpad and brains were harvested at 0, 4, 7 and 28 dpi. **(B)** C57BL/6 mice H&E data images in hippocampus (5x) and cortex (10x). **(C)** C57BL/6 mice H&E data dentate gyrus (DG) blade length quantification, H&E hippocampus area measurement and H&E cortex positive cell count detection, n=3-4. **(D)** Corpus callosum H&E with quantification (10x, 20x). **(E)** TUNEL images of mouse hippocampus and cortex 15x. Followed by quantification. TUNEL-positive cells were manually counted in a defined region of interest (ROI) in the hippocampus/cortex. **(F)** 40X immunofluorescence of GFAP in corpus callosum for UI and D28. N=3-4. Data represents the mean ± SD ^∗^p < 0.0332. The experiment was independently repeated twice with similar results; representative data are shown.

C57BL/6 adult mice were infected with ZIKV (MR766 strain; 10^6 PFU) via footpad injection to assess the impact of ZIKV on neuropathogenesis in the adult brain. One day before infection, IFNAR antibody, an IFN-A receptor antagonist, was administered to enable efficient viral infection, as ZIKV NS5 protein cannot effectively inhibit STAT2 in mice (33). Brain tissues were collected from uninfected (UI) and infected mice at 4-, 7-, and 28-days post-infection (dpi) (**Figure 1A**). As ZIKV targets memory-related regions, particularly the hippocampus and cortex, histological analysis focused on these areas. H&E staining revealed a reduction in the length of the dentate gyrus blade in the hippocampus at 4 dpi, returning to near baseline by 7 and 28 dpi (**Figures 1B, C**). Additionally, the hippocampal area expanded at 28 dpi, and notable immune cell infiltration was observed in the cortex (**Figures 1B, C**).

Furthermore, immune cell infiltration was observed in the corpus callosum at 4 dpi (**Figure 1D**). To determine if this immune response was linked to neuronal cell death, we performed a TUNEL assay at multiple time points. At 4 dpi, TUNEL-positive cells were detected in both the hippocampus and cortex (**Figure 1E**), indicating apoptosis in these memory-related regions. No TUNEL-positive cells were observed at later time points (**Supplementary Figure 1C**), suggesting a subsidence of apoptosis over time. However, other forms of cell death may contribute to chronic brain damage. Additionally, because astrocytes are key modulators for the BBB, we wanted to briefly identify any changes in these cell types for our mice (21). Astrocyte marker GFAP was reduced in the corpus callosum of the ZIKV-infected mice (**Figure 1F**). These findings demonstrate acute and chronic brain pathology in adult immunocompetent mouse models following ZIKV infection.

### 3.2 ZIKV RNA and inflammatory genes are detectable in the brain during acute infection

**Figure 2:**
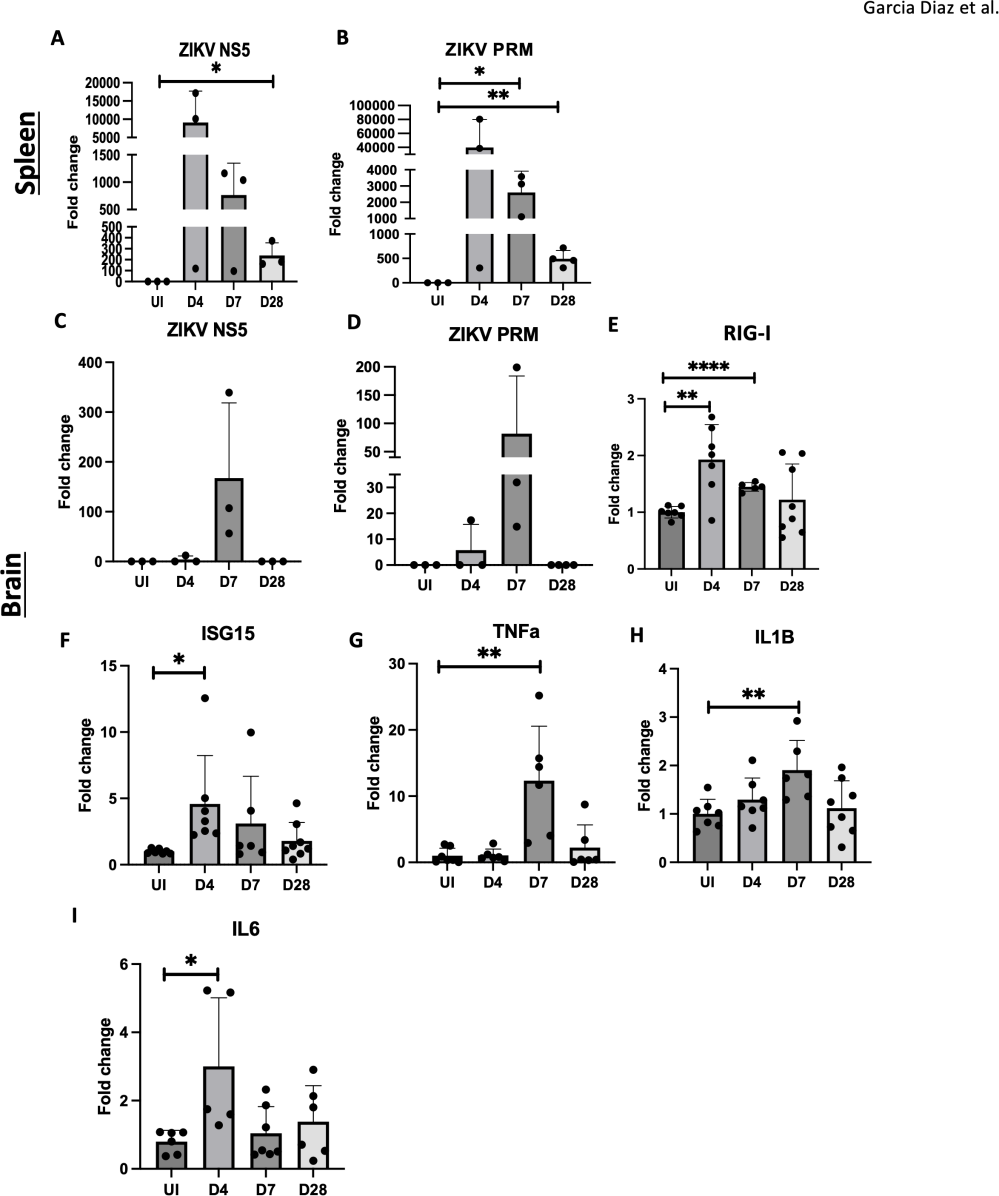
Inflammation observed in ZIKV infected adult brain. **(A, B)** RT-qPCR of ZIKV NS5, PrM in C57BL/6 mice spleens. **(C–I)** RT-qPCR of ZIKV NS5, PrM, RIG-I, ISG15, TNFa, IL1B and IL6 and in mouse brain for UI, D4, D7 and D28. N=3-8. Data represents the mean ± SD ^∗^p < 0.0332, ^∗∗^p < 0.0021, and ^∗∗∗∗^p < 0.0001. The experiment was independently repeated twice with similar results; representative data are shown.

To assess ZIKV presence and the inflammatory response, we performed RT-qPCR for ZIKV RNA and inflammatory gene markers. High levels of ZIKV RNA (*NS5*, *PRM*) were detected in the spleen at 4 dpi, with a decline over time (**Figures 2A, B**), confirming viral infection in adult mice. In contrast, ZIKV RNA was only detectable in the brain at 7 dpi (**Figures 2C, D**), despite the observed pathology at 4 dpi (**Figure 1B**). We then analyzed the expression of *RIG-I* and *ISG15*, genes induced by viral RNA as pathogen-associated molecular patterns (PAMPs). Notably, *RIG-I* and *ISG15* expression were elevated at 4 dpi and 7 dpi, suggesting viral RNA presence on day 4, despite being below the RT-qPCR detection threshold (**Figures 2E, F**).

Additionally, proinflammatory cytokine genes, IL1β and TNFα, were elevated at 7 dpi, and IL6 was upregulated on 4 dpi, in brain tissue (**Figures 2G–I**). IL6 and TNFα gene levels remained elevated in the hippocampus at 4, 7, and 28 dpi (**Supplementary Figures 1A, B**). These results indicate that viral RNA, rather than viral replication alone, triggers PAMP signaling and the innate immune response, contributing to neuroinflammation and potential CNS damage.

### 3.3 Increased expression of microglial inflammatory genes and microglial activation following ZIKV infection

**Figure 3:**
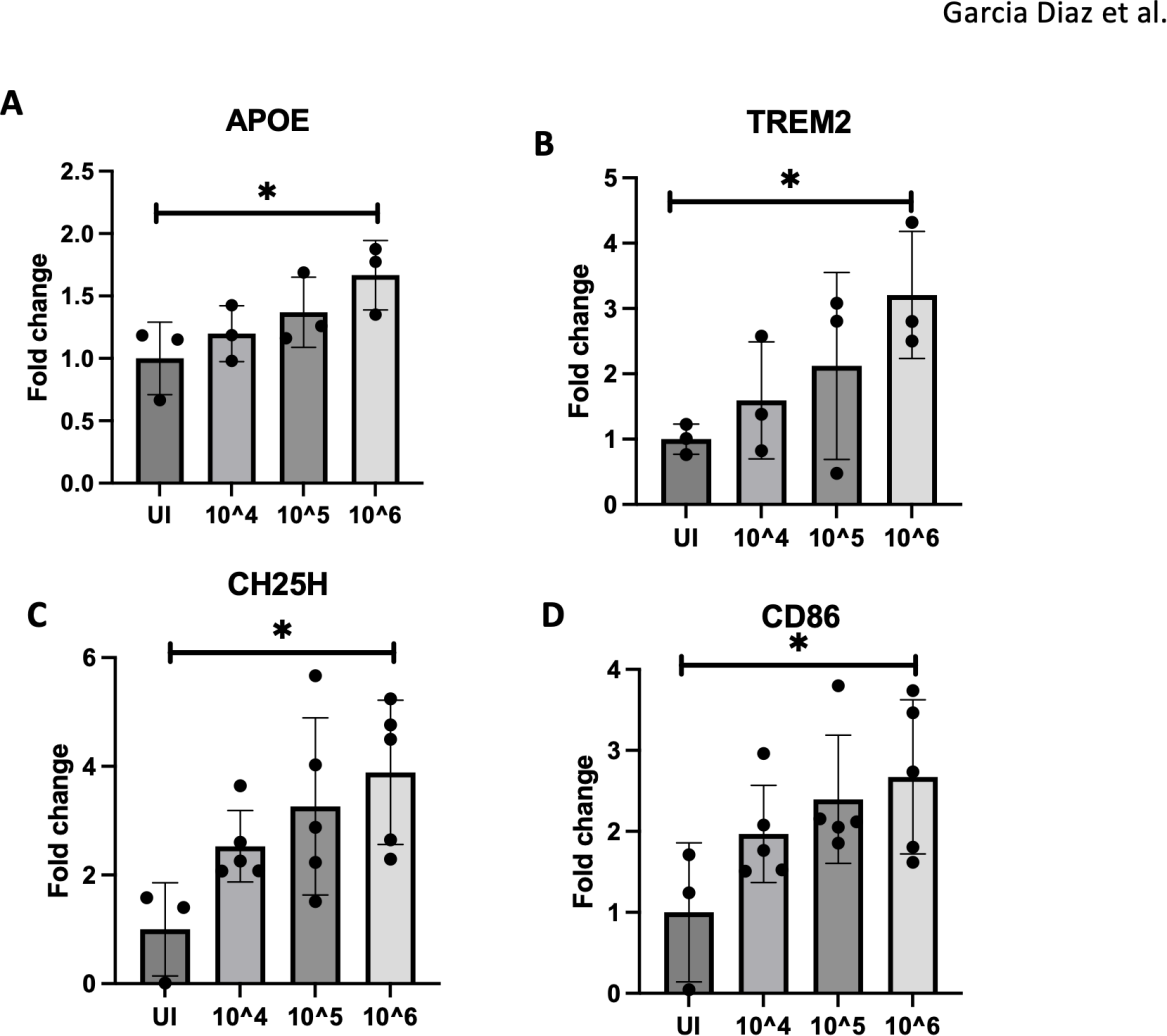
10^6 PFU viral dose results in pronounced microglial activation. **(A–D)** RT-qPCR of APOE, TREM2, CH25H and CD86 expression with varying doses of ZIKV (UI, 10^4,10^5,10^6) for 4dpi. N=3-5. Data represents the mean ± SD ^∗^p < 0.0332. The experiment was independently repeated twice with similar results; representative data are shown.

Disease-associated microglia (DAMs) are prominent in Alzheimer’s disease (AD) and play a role in exacerbating brain pathology (23, 30). To determine if DAMs are involved in ZIKV-related brain damage, mice were infected with varying doses of ZIKV (uninfected, 10^4, 10^5, and 10^6 PFU). Brain tissues were then harvested at 4 dpi for DAMs gene analysis. Notably, microglial activation markers such as APOE and TREM2, associated with DAMs, increased with higher viral doses (**Figures 3A, B**). Inflammatory markers CH25H and CD86 also peaked at 10^6 PFU, indicating a dose-dependent inflammatory response (**Figures 3C, D**). CH25H, an enzyme that converts cholesterol to 25-HC, is known to trigger proinflammatory cytokine production (20). Our prior studies showed CH25H upregulation in microglia following ZIKV infection (34). Of note, lower viral doses showed similar trends without statistical significance.

**Figure 4:**
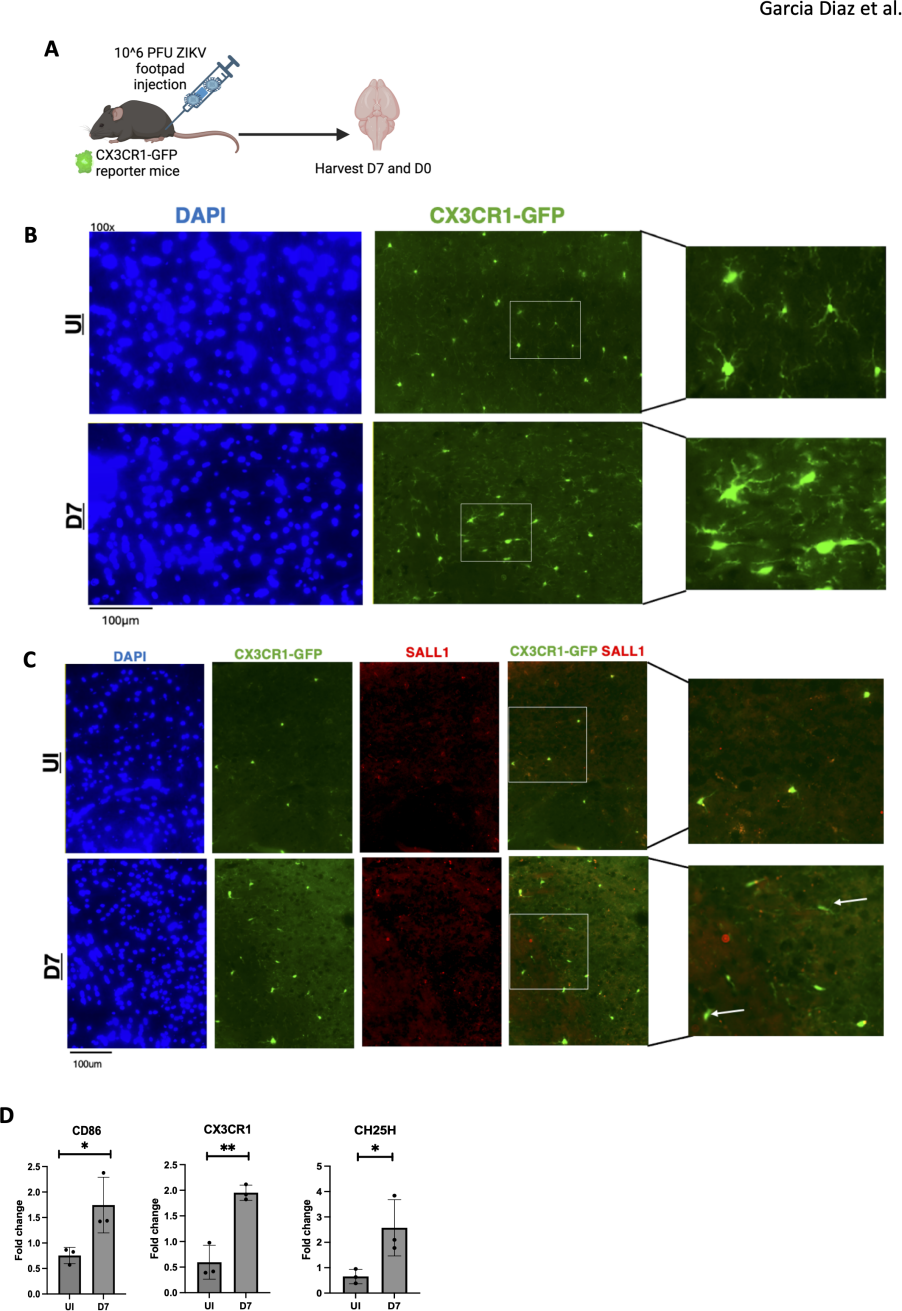
ZIKV results in activated microglia with a change in morphology. **(A)** CX3CR1-GFP mice were injected with ZIKV at 10^6 PFU via footpad, brains were harvested at 0 and 7dpi. **(B)** 100X immunofluorescence imaging of mouse cortex staining for DAPI and CX3CR1. **(C)** 100X immunofluorescence of mouse cortex to assess microglial morphology using sall1 and CX3CR1-GFP. **(D)** PCR data for CD86, CX3CR1 and CH25H genes, N=3. Data represents the mean ± SD ^∗^p < 0.0332, ^∗∗^p < 0.0021. The experiment was independently repeated twice with similar results; representative data are shown.

We next assessed microglial morphology in CX3CR1-GFP reporter mice (**Figure 4A**). Widefield fluorescence microscopy was utilized to localize specific brain regions before examining areas of interest. At 7 dpi, ZIKV-infected mice exhibited altered microglial morphology, indicative of an activated, stressed state (**Figure 4B**) (19). The extended microglia morphology suggests activation, likely in response to viral PAMPs or signals from infiltrating cells (**Figure 4B**). The expression of sall1 confirms that CX3CR1-GFP mice are labeling microglia, as sall1 is a common microglial marker (35). Additionally the lack of sall1 on some CX3CR1-GFP cells suggests a downregulation of the homeostatic marker for microglia, further indicating microglial activation (**Figure 4C**) (35).

Moreover, RT-qPCR of brain tissue showed elevated *CD86, CX3CR1*, and *CH25H* expression in infected mice at 7 dpi compared to controls, consistent with microglial activation (**Figure 4D**). Notably, in Alzheimer’s, CH25H inhibition reduces neurodegeneration and neuroinflammation, particularly in the hippocampus (PS19 mice) (36). Given CH25H’s role in lipid metabolism, we examined lipid deposits in ZIKV-infected brains. Oil-Red staining revealed lipid accumulation in the hippocampus of infected mice at 7 dpi (**Supplementary Figure 2**), indicating dysregulated lipid metabolism. These findings suggest that activated microglia may contribute to neuroinflammation through the production of neurotoxins, and targeting this inflammation could present therapeutic opportunities.

### 3.4 Transcriptomic analysis reveals distinct gene expression patterns in acute versus chronic ZIKV infection, with chronic inflammation persisting in the hippocampus

**Figure 5:**
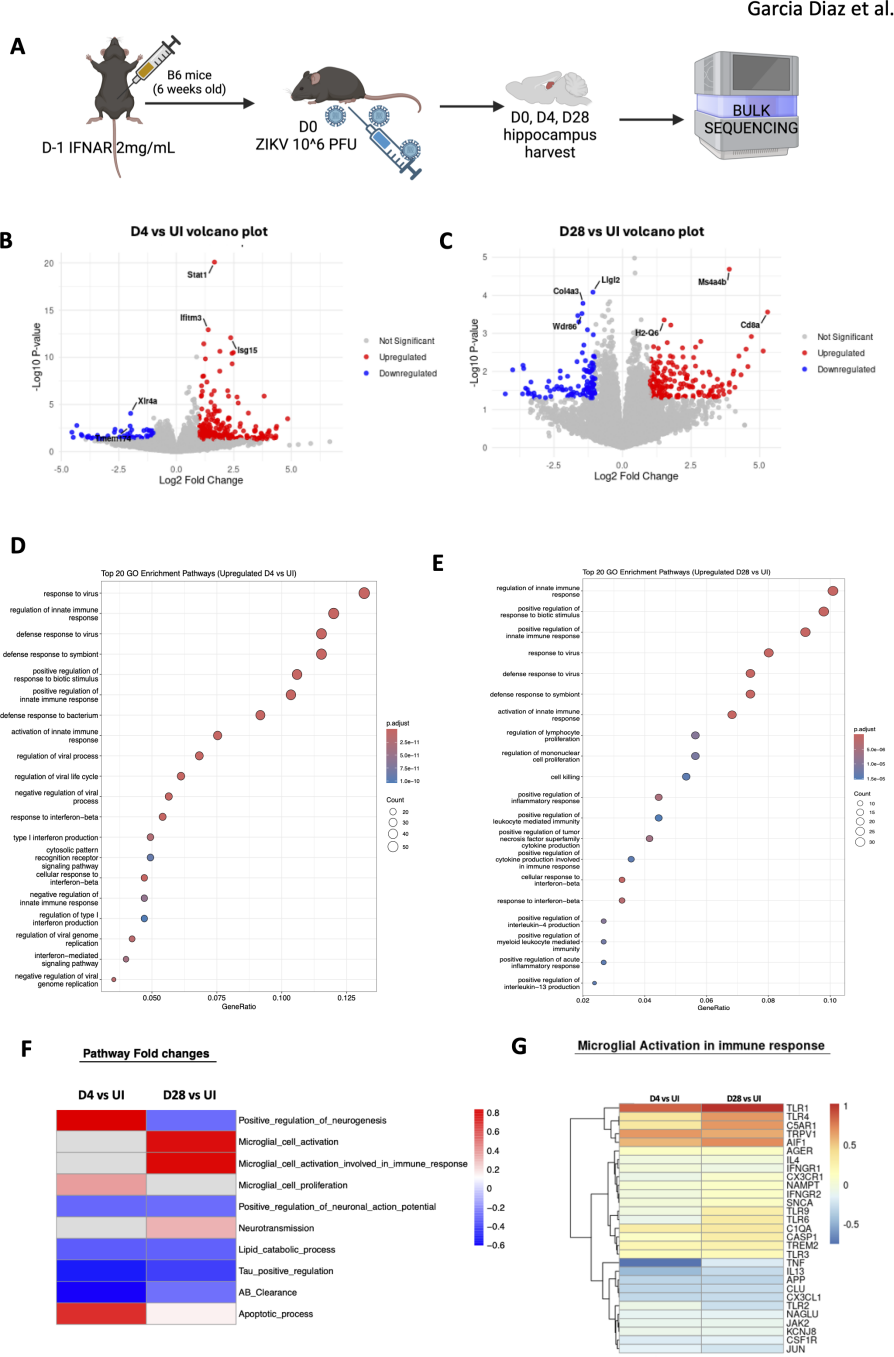
Bulk sequencing of mouse hippocampus shows chronic pathology. **(A)** Mice hippocampal sections were harvested and sequenced. **(B, C)** Volcano plot showing the top three upregulated and downregulated genes for D4 vs. UI. **(C)** shows D28 vs UI volcano plot for the top three upregulated and downregulated genes. Red indicates upregulated with a positive log2fold change value (>1) and blue indicates downregulated with a negative log2fold change value (<1). Grey is non-significant genes. **(D)** Gene expression differences comparing D4 to UI visualizing the top most upregulated genes in the D4 group. **(E)** Gene expression differences comparing D28 to UI visualizing the top most upregulated genes in the D28 group. **(F)** Heatmap representing fold changes of GO pathways of D4 vs UI and D28 vs UI mouse hippocampi. Blue=downregulated and Red=upregulated. N=3 per condition **(G)** Specific genes in microglial activation involved in immune response pathway that are altered.

To investigate ZIKV-induced chronic pathology, we conducted bulk RNA sequencing on mouse hippocampus samples, comparing infected mice at 4 and 28 dpi to uninfected controls (**Figure 5A**). We identified genes upregulated and downregulated at both time points relative to uninfected controls (**Supplementary Figure 3A**). While the profiles at 4 and 28 dpi differed, 151 genes were upregulated, and 138 were downregulated in acute and chronic stages of infection. At 4 dpi, genes such as *Stat1, Ifitm3*, and *Isg1*, primarily involved in IFN signaling, were upregulated, whereas at 28 dpi, genes like *Cd8a, Ms4a4b, H2-Q6*, which are associated with T cell responses were notably elevated (**Figures 5B, C**). Among the top 10 genes shared between both time points, upregulated pathways included IFN signaling and pathogen recognition with higher fold observed at 4 dpi compared to 28 dpi, while downregulated genes were predominantly related to cellular stress responses, showing a similar level of reduction at both time points (**Supplementary Figures 3B, C**). This downregulation may reflect the immune system’s attempt to control inflammation, which could otherwise exacerbate pathology.

Gene ontology (GO) enrichment analysis confirmed these findings, revealing upregulation of viral response genes and continued activation of IFN signaling at 4 and 28 dpi (**Figures 5D, E**). IFN signaling is critical for viral clearance, and prior studies have highlighted its importance in ZIKV infection in adult brains (6). At 4 dpi, the immune system was actively engaged in the hippocampus, with heightened viral response genes (**Figure 5D**). By D28, the viral response persisted, with increased expression of genes involved in cytotoxicity, indicating sustained immune activation (**Figure 5E**). Chronic inflammation was evident, with inflammatory pathways still upregulated 4 weeks post-infection. Notably, the “regulation of mononuclear cell proliferation” pathway was upregulated, suggesting infiltrating immune cells, such as monocytes, may contribute to observed pathology. In contrast, apoptotic cell clearance pathways were downregulated at 4 dpi, potentially exacerbating inflammation in the upregulated gene sets (**Supplementary Figure 3D**). At 28 dpi, extracellular matrix (ECM)-related genes were similarly downregulated, a change that may hinder cell repair and proliferation (**Supplementary Figure 3E**), consistent with previous studies linking ECM alterations to impaired neuronal regeneration after viral infections (37).

We then extracted genes from GO categories and compared these results with RT-qPCR and histology data with fold-change values between infected and uninfected groups visualized in a heatmap (**Figure 5F**). Notably, apoptotic processes were upregulated in both acute (4 dpi) and chronic (28 dpi) infection-though more pronounced at 4 dpi. In contrast, lipid catabolism was downregulated in both phases, which is significant as brain lipid alterations are implicated in neurodegenerative diseases like Alzheimer’s. Moreover, *Tau* and *amyloid-beta* (Aβ) genes were downregulated at both time points, suggesting potential memory-related deficits in the mice, as these proteins are critical in Alzheimer’s pathology.

Interestingly, neurotransmission genes were upregulated at 28 dpi, indicating that neurons may initially attempt to recover from infection but ultimately succumb to chronic inflammation, possibly contributing to neurocognitive dysfunction. Additionally, microglial cell proliferation was upregulated at 4 dpi, while their activation was more pronounced at 28 dpi, suggesting that microglia play a central role in driving chronic inflammation in the hippocampus (**Figures 5F, G**). This comprehensive analysis underscores the long-lasting effects of ZIKV infection in the hippocampus, revealing both acute and chronic immune responses that likely contribute to persistent neuroinflammation and potential cognitive deficits.

### 3.5 Monocyte infiltration into brain tissue was detectable at 7 days after ZIKV infection

Although high amounts of pathogens like ZIKV are not present in the adult brain, they can still trigger inflammation potentially through peripheral effects or blood-brain barrier (BBB) disruption, enabling immune cells to enter the CNS (32). Monocytes have been shown to infiltrate the brain in immunocompromised mouse models, and clinical studies have reported similar infiltration of mononuclear cells in adults (12, 38). To investigate the role of monocyte infiltration in ZIKV-induced neuroinflammation and CNS damage, we examined monocyte migration into the brain following infection.

**Figure 6:**
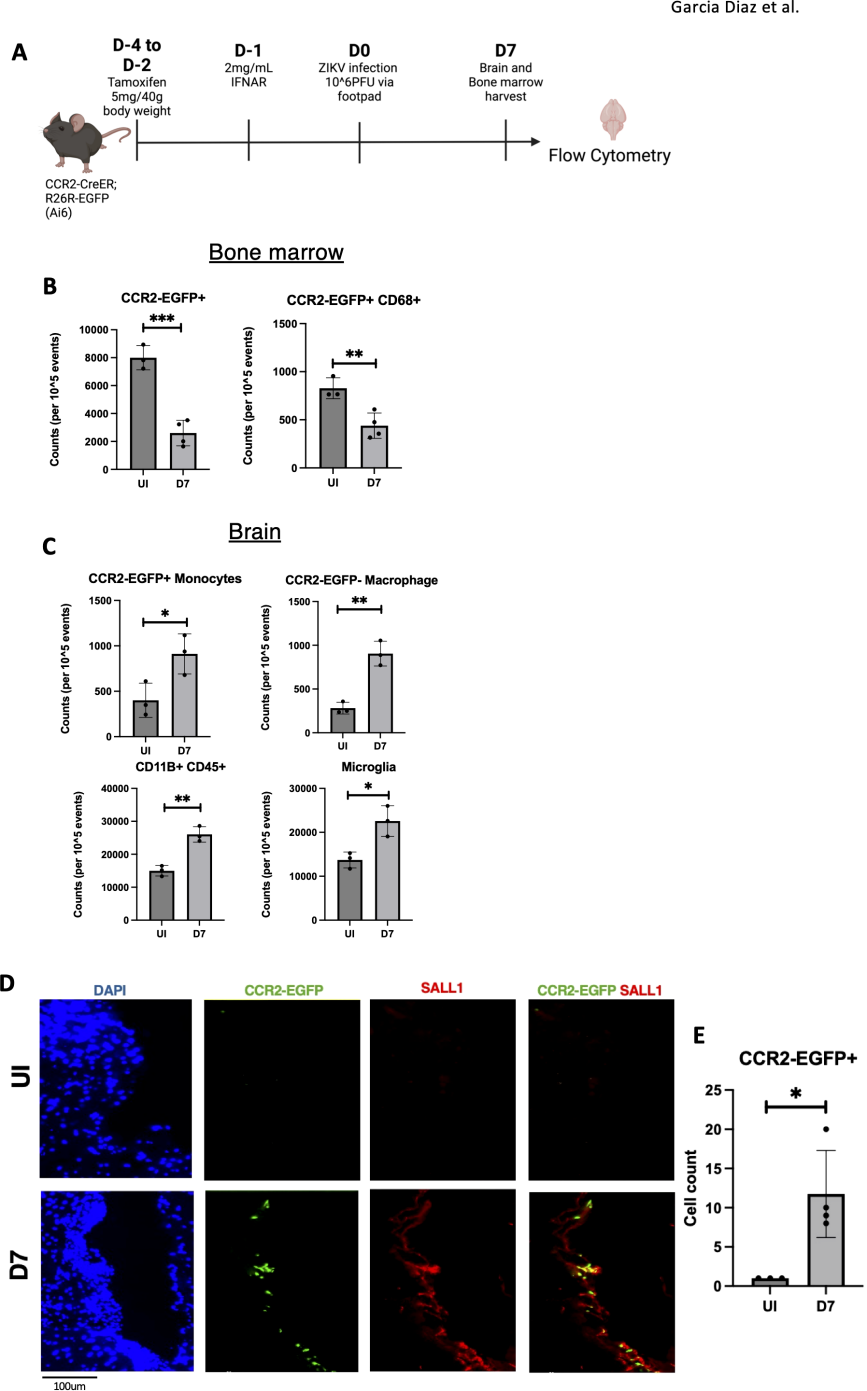
Infiltrating monocytes seen in ZIKV mice brains at 7dpi. **(A)** CCR2-CreER;R26R-EGFP (Ai6) mice were given tamoxifen for 3 days before infected with ZIKV and harvested. **(B)** Quantification of flow data for Bone marrow cells (CCR2-EGFP+, CCR2-EGFP+ CD68+) **(C)** Quantification of flow data for Brain cells (CD11B+CD45+, CD45int CD11B+ (Microglia), CCR2-EGFP+, CCR2-EGFP-). **(D, E)** Immunofluorescence of CCR2-CreER-R26R-EGFP mice for EGFP and sall1 expression in choroid plexus region with quantification. N=3-4. Data represents the mean ± SD ^∗^p < 0.0332, ^∗∗^p < 0.0021, and ^∗∗∗^p < 0.0002.

To track monocyte-derived cells, we used CCR2-creER-R26R-EGFP (Ai6) mice, which express EGFP in monocytes and their progeny, even after CCR2 is downregulated in the brain (39). This allows us to trace the migration of these cells despite changes in their surface markers. Mice were treated with tamoxifen (5 mg/40 g body weight) for 3 days, followed by IFNAR antibody administration one day before ZIKV footpad infection. At 7 dpi, mice were sacrificed, and cells isolated from brain tissue were analyzed by flow cytometry to track monocyte-derived cells (**Figure 6A**). In parallel we harvested bone marrow to assess potential monocyte migration to the brain by quantifying changes in monocyte-derived cell populations. Flow cytometry of bone marrow revealed a significant reduction in CCR2-EGFP+ monocyte-derived cells (**Figure 6B**), suggesting migration of these cells to the brain or cell death due to viral infection. A concurrent decrease in CD68+ monocytes supported the idea of monocyte migration or cell loss (**Figure 6B**).

In the brain, flow cytometry revealed an increased myeloid cell population (CD45+ CD11B+) at 7 dpi, indicating immune cell infiltration (**Figure 6C**). Further gating analysis showed an increase in microglia, suggesting microglial activation (34). Importantly, we detected an increase in CCR2-EGFP+ monocyte-derived cells and CCR2-EGFP- macrophages. (**Figure 6C**). Immunofluorescence of these same mice confirmed monocyte-derived cells near the choroid plexus at 7 dpi (**Figures 6D, E**). Overall, these findings demonstrate significant monocyte infiltration and microglial activation in the brain by 7 dpi, suggesting a role for infiltrating monocytes in driving neuroinflammation.

### 3.6 ZIKV infection drives proinflammatory monocytes and CCR2 inhibition mitigates neuroinflammation

**Figure 7:**
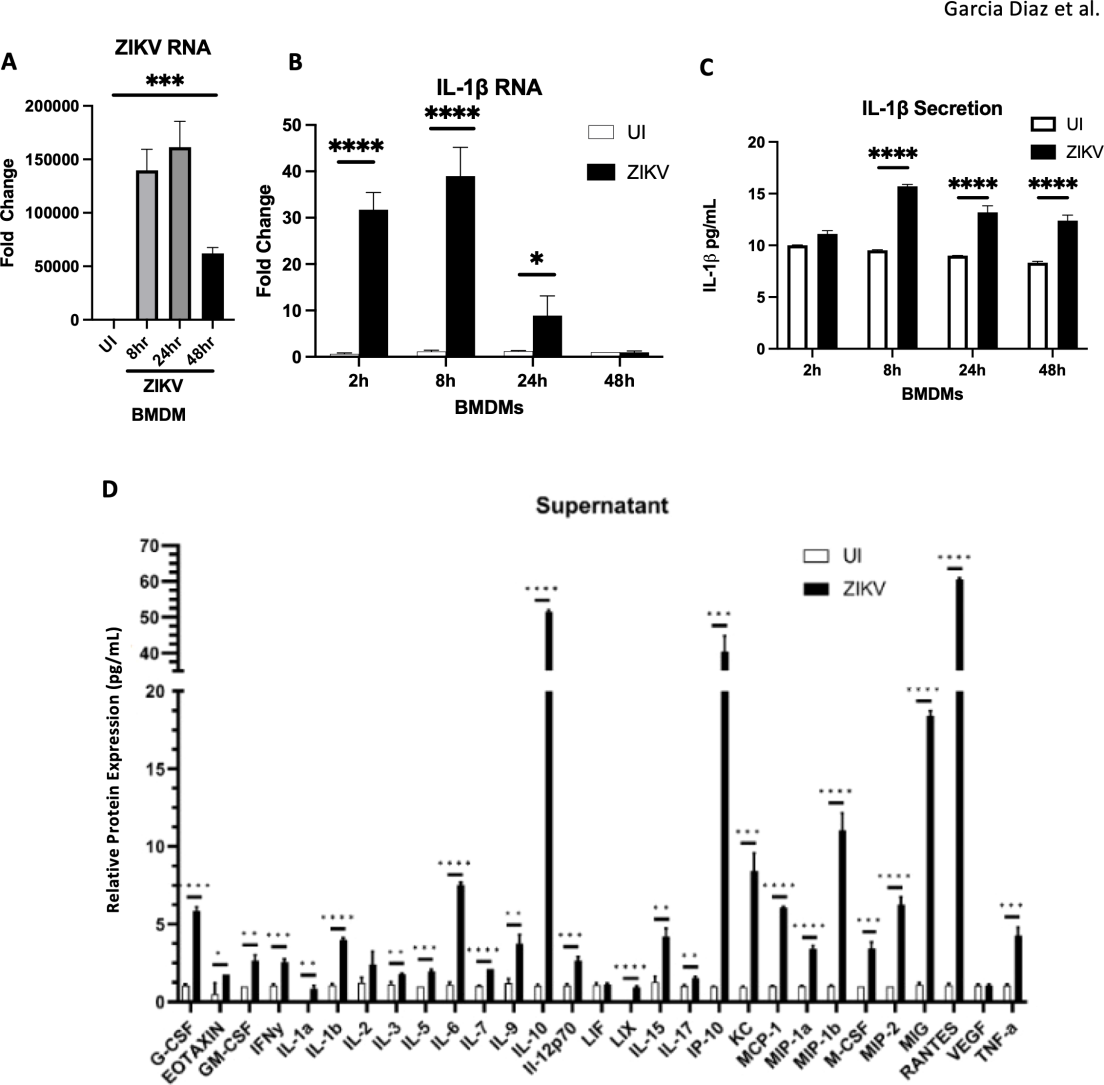
BMDMs infected by ZIKV and lead to the release of inflammatory cytokines **(A, B)** RT-qPCR for ZIKV RNA, and *IL-1β* mRNA. **(C)** ELISA for IL1B secretion. **(D)** Mouse 32 Plex Luminex assay was run by the UVA Flow Cytometry Core Facility. Data represents the mean ± SD ^∗^p < 0.0332, ^∗∗^p < 0.0021, and ^∗∗∗^p < 0.0002, ^∗∗∗∗^p < 0.0001. The experiment was independently repeated twice with similar results; representative data are shown.

To assess ZIKV’s ability to drive monocyte inflammatory responses, we measured IL1β production in bone marrow-derived macrophages (BMDMs) following infection. BMDMs support ZIKV replication (**Figure 7A**), and exhibit increased *IL1β* RNA and protein levels post-infection (**Figures 7B, C**). IL1β production, induced by ZIKV, is linked to neuronal cell death (40). Cytokine/chemokine array analysis revealed that BMDMs secrete various cytokines and chemokines, including MCP-1, which are involved in monocyte recruitment (**Figure 7D**). These findings suggest that ZIKV infection promotes inflammatory factor release, triggering monocyte infiltration which may contribute to *in vivo* neuroinflammation.

**Figure 8:**
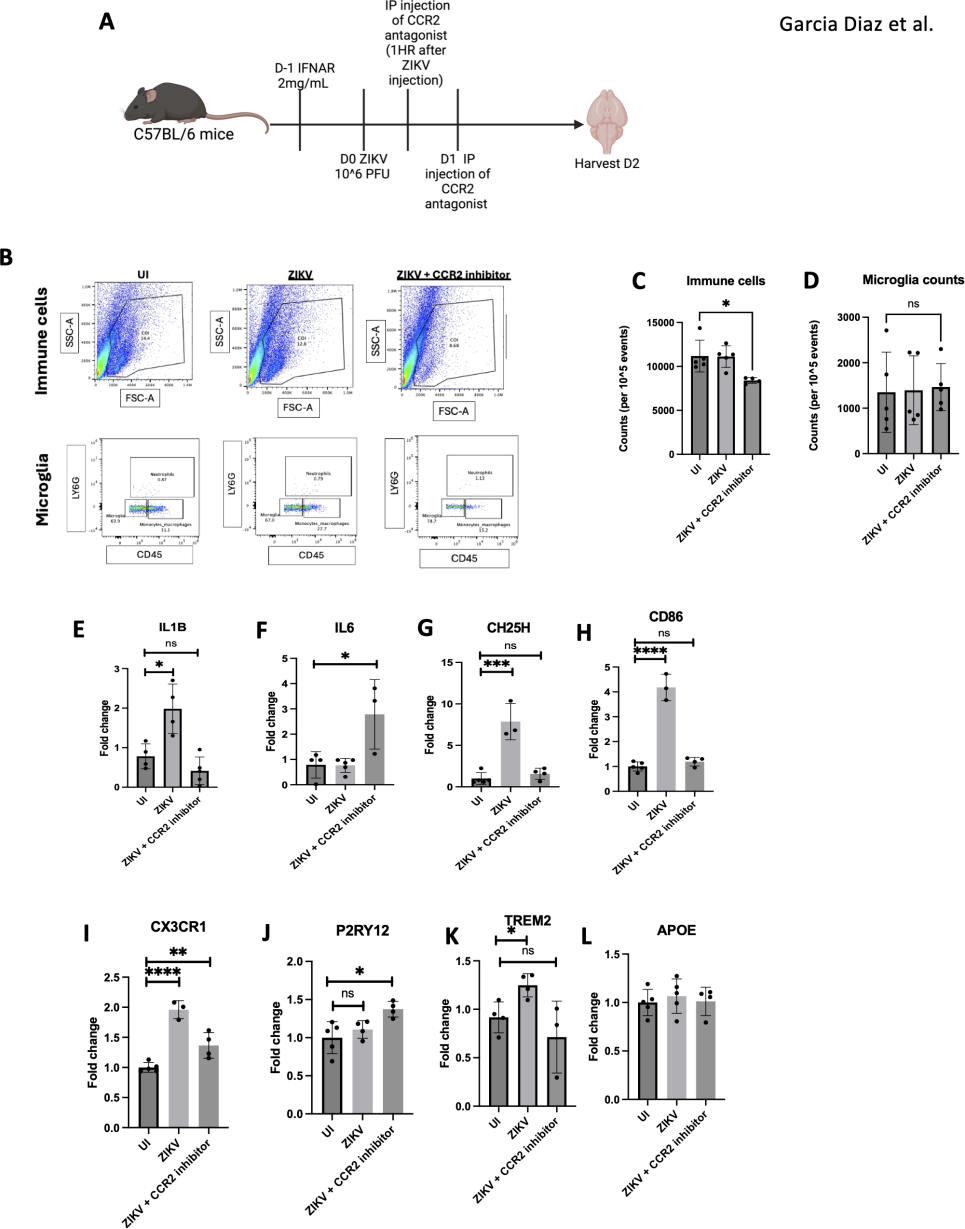
Monocyte inhibition ameliorates ZIKV neuroinflammation. **(A)** C57BL/6 mice were injected with ZIKV then CCR2 inhibitor at 1 hpi and 24hpi before harvesting at 2 dpi. **(B)** FACs of total immune cell and microglia. **(C)** FACs quantification of cell counts and microglia **(D)**. **(E–L)** RT-qPCR of half brain regions for IL1B, IL6, CH25H, CD86, CX3CR1, P2RY12, TREM2 and APOE. N=3-5. Data represents the mean ± SD ^∗^p < 0.0332, ^∗∗^p < 0.0021, and ^∗∗∗^p < 0.0002.

To explore the role of monocytes in neuroinflammation and CNS damage, we administered the CCR2 inhibitor RS-102895. CCR2 mediates monocyte chemotaxis toward inflammation sites and acts as a receptor for MCP-1 (CCL2) (41). Mice were treated with 10 mg/kg of CCR2 inhibitor one hour and 24 hours after ZIKV infection, followed by brain harvesting 48 hours post-infection (**Figure 8A**). Flow cytometry analysis showed a significant reduction in overall immune cell counts in ZIKV-infected mice treated with the CCR2 inhibitor, confirming the effective inhibition of monocyte recruitment (**Figures 8B, C**). Notably, microglial cell counts remained unchanged, indicating that the inhibitor did not affect microglia (**Figure 8D**).

We observed a reduction in neuroinflammation in the CCR2 inhibitor-treated mice by decreased expression of *IL1β, CH25H*, and *CD86* in the brain. Additionally, *CX3CR1* expression, a microglial activation marker, was decreased, suggesting reduced microglial activation (42, 43). The upregulation of *P2RY12*, associated with anti-inflammatory microglia, further supports this finding (44). Lastly, *TREM2*, a known DAM marker, was reduced, indicating a decrease in disease-associated microglia. The increase in *IL6* observed in the CCR2 inhibitor-treated group could indicate a compensatory mechanism to ameliorate virus induced pathology (**Figures 8E–L**). The combined decrease in *CX3CR1*, increase in *P2RY12*, and reduction in *TREM2* suggests that CCR2 inhibition attenuates neuroinflammation by modulating microglial gene expression.

## 4 Discussion

In this report, we demonstrate that ZIKV infection induces neuroinflammation in the adult brain, characterized by microglial activation and monocyte cell infiltration. A recent study found that immunocompromised mice with intracerebral ZIKV infection show activated microglia and altered transcriptomic profiles in the cerebral cortex (30). However, this study is limited due to the unnatural mode of infection as well as the deficiency of host immunity. Using a model combining transient IFNAR blockade with footpad ZIKV infection, our study better mimics natural infection and pathogenesis in adults. Unlike intracerebral injection or immunodeficient models, this approach did not result in mouse mortality, suggesting less aggressive neuroinvasion. Despite low viral burden in the brain parenchyma, we observed infiltrating monocytes and activated microglia, as seen through altered microglial morphology, increased microglial cell counts, and elevated expression of microglial inflammatory genes in ZIKV-infected brains one-week post-infection. Additionally, some microglia in CX3CR1-GFP mice did not express sall1, suggesting microglial activation as sall1 downregulation is associated with this process (35). Understanding these key factors driving brain pathology is crucial for developing therapeutic strategies for ZIKV-associated neurological deficits.

Our studies show elevated *IL1β* and *TNFα* gene expression up to day 7, likely driven by ZIKV infection and apoptosis in brain tissue. *In vitro* studies on ZIKV-infected mouse BMDMs revealed increased *IL1β*, linked to inflammatory cell death (6). Notably, neuroinflammation was observed on day 4, even in the absence of ZIKV RNA. Interestingly, the RNA sensor *RIG-I* was upregulated on days 4 and 7 (**Figure 2**), suggesting persistent immune activation. Although viral RNA was undetectable in the brain at later timepoints, spleen analysis may clarify the sustained neuroinflammation observed at day 28. As demonstrated in studies of SARS-CoV and TBEV (45, 46), peripheral infection, such as in the spleen, could indirectly drive neuroinflammation. Further investigation with more sensitive assays will clarify whether residual viral RNA or peripheral immune signals drive inflammatory response in the brain, along with luminex or ELISA studies in brain tissue to validate our findings.

Bulk sequencing revealed upregulation of apoptosis, chronic inflammation, and mononuclear cell infiltration in the hippocampus (**Figure 5**), a key region involved in memory formation (47). If brain inflammation is not treated ongoing inflammation can lead to encephalitis associated with a mortality rate of 5.6–5.8% and altered mental status (48). Prolonged inflammatory activation was seen in our mouse model, along with microglial activation being upregulated during chronic infection. ZIKV has been linked to cognitive deficits and altered mental status in humans; this underlying pathology may, in part, stem from neuroinflammation. The sustained increase in IFN-related genes in our sequencing data suggests that our transient blockade of IFN signaling is indeed temporary, supporting its critical role in viral response. The upregulation of *CH25H* gene, involved in cholesterol synthesis, was observed in ZIKV-infected brain. Additionally, lipid deposits were observed in the brain following ZIKV infection. These findings suggest the need for future investigations into lipid accumulation in ZIKV-associated cognitive dysfunction, given the role of lipids in Alzheimer’s disease progression (49).

Notably, monocyte recruitment was increased during ZIKV infection, and appeared to drive inflammatory responses in the brain (25). Blocking monocyte infiltration reduced the expression of microglial activation genes. This is consistent with findings from other neurotropic viruses that infiltrating monocytes play a role in exacerbating neuroinflammation and activating microglia (25, 50). Notably, inflammation and cell death occurred even when viral RNA was undetectable, implicating blood–brain barrier (BBB) disruption. Reduced astrocyte numbers and downregulation of ECM-related genes in the hippocampus suggest BBB compromise at 28 dpi, potentially facilitating monocyte entry. Future studies using BBB permeability assays are necessary to confirm this. Monocyte infiltration, coupled with microglial expansion (**Figure 6**) and supported by inhibitor studies (**Figure 8**), implicates monocytes as key drivers of neuroinflammation. Cells co-stained for sall1 and CCR2-EGFP (**Figure 6D**, arrow) may indicate monocyte-to-microglia transition, though further validation is needed. Prolonged CCR2 inhibition studies could help define its role in sustaining inflammation and microglial activation during chronic ZIKV infection.

**Figure 9:**
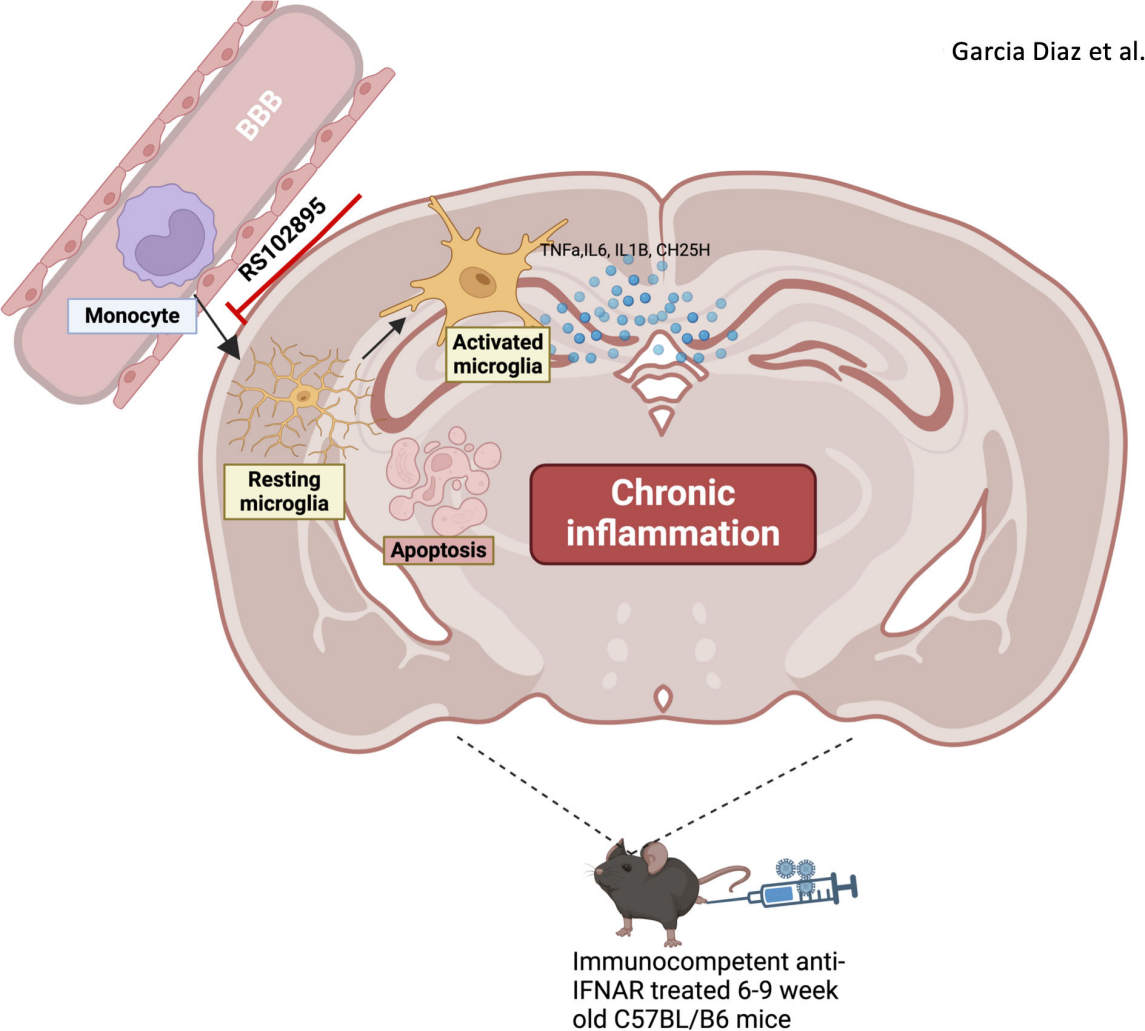
Infiltrating monocytes heighten brain neuroinflammation by activating microglia following ZIKV. Monocytes infiltrate brain parenchyma after zika virus infection in the immunocompetent adult brain. Microglia become activated following zika virus infection and inflammation persists up to D28. The inhibition of monocytes reduces neuroinflammation in the immunocompetent adult brain.

In conclusion, ZIKV infection in immunocompetent adult mice resulted in brain inflammation, marked by monocyte infiltration and microglia activation. These microglia are likely activated by infiltrating monocytes during infection, with brain pathology persisting 28 days post-infection (**Figure 9**). Our findings advance the understanding of ZIKV-driven CNS inflammation and its potential long-term neurological impact.

## Data Availability

The original contributions presented in the study are included in the article/**Supplementary Material**. Additionally Bulk RNA seq data produced from this study is accessible through NCBI. This data can be found here: BioProject ID: PRJNA1282613 with BioSample accessions: SAMN49653677, SAMN49653678, SAMN49653679, SAMN49653680, SAMN49653681, SAMN49653682, SAMN49653683, SAMN49653684, SAMN49653685. Further inquiries can be directed to the corresponding author.

## Supplementary material

Supplementary Figure 1 Inflammation seen in hippocampus following ZIKV infection. **(A, B)** IL6 and TNFa RNA detection via RT-qPCR in brain regions. **(C)** TUNEL assay for D7 and D28 for hippocampus and cortex. UI, uninfected; BrS, brain Stem; CX, cortex; CB, cerebellum; H, hippocampus. Data represents the mean ± SD ^∗^p < 0.0332, ^∗∗^p < 0.0021.

Supplementary Figure 2 Lipid detection observed at 7dpi. **(A)** Oil red staining of brain tissue with quantification **(B)**. Data represents the mean ± SD ^∗^p < 0.0332.

Supplementary Figure 3 Bulk sequencing reveals diverse transcriptomic profile with chronic effects. **(A)**: Left Venn diagram depicts the counts of upregulated genes in D4 and D28 compared to UI that have a p value < 0.05 and a log2foldchange >0. Right Venn diagram shows downregulated genes in D4 and D28 compared to UI that have a p value < 0.05 and a log2foldchange < 0. **(B, C)** Top 10 genes found to be upregulated/downregulated in both D4 and D28 groups, with a p value < 0.05. (n=3). **(D)** Gene expression differences comparing D4 to UI visualizing the top most downregulated genes in the D4 group. **(E)** Gene expression differences comparing D28 to UI visualizing the top most downregulated genes in the D28 group.

Supplementary Figure 4 Gating strategy for CCR2-creER-R26R EGFP (Ai6) mice for brain **(A)** and bone marrow **(B)**.
