# Supplementary figures and images for: Zika virus induces monocyte recruitment in the immunocompetent adult brain driving chronic inflammation
Source: Front Immunol. 2025 Jul 4;16:1597776. doi: 10.3389/fimmu.2025.1597776 (PMC12271104; doi:10.3389/fimmu.2025.1597776)

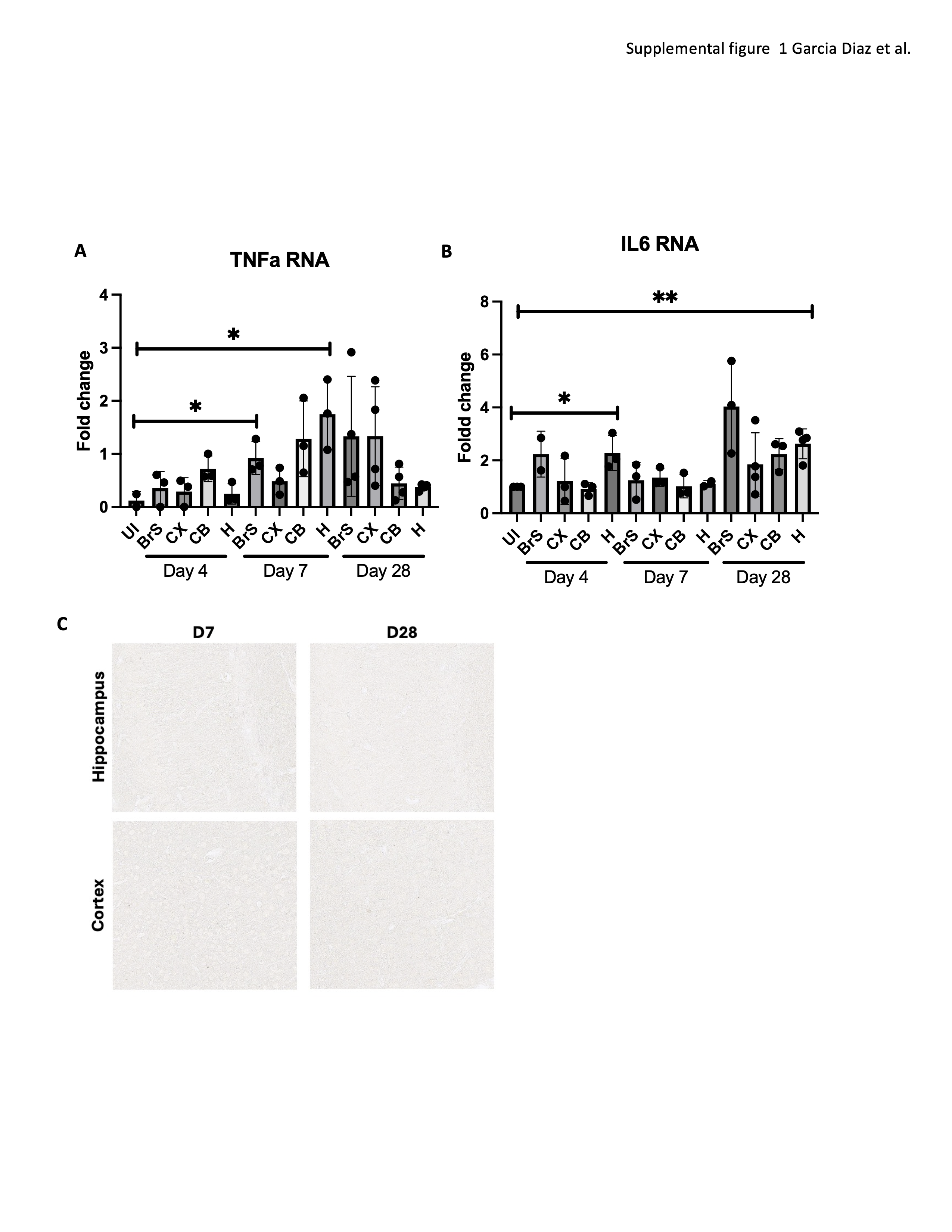

Supplement: Supplementary Figure 1 — Inflammation seen in hippocampus following ZIKV infection. (A, B) IL6 and TNFa RNA detection via RT-qPCR in brain regions. (C) TUNEL assay for D7 and D28 for hippocampus and cortex. UI, uninfected; BrS, brain Stem; CX, cortex; CB, cerebellum; H, hippocampus. Data represents the mean ± SD ∗p < 0.0332, ∗∗p < 0.0021. [file Image1.tiff]

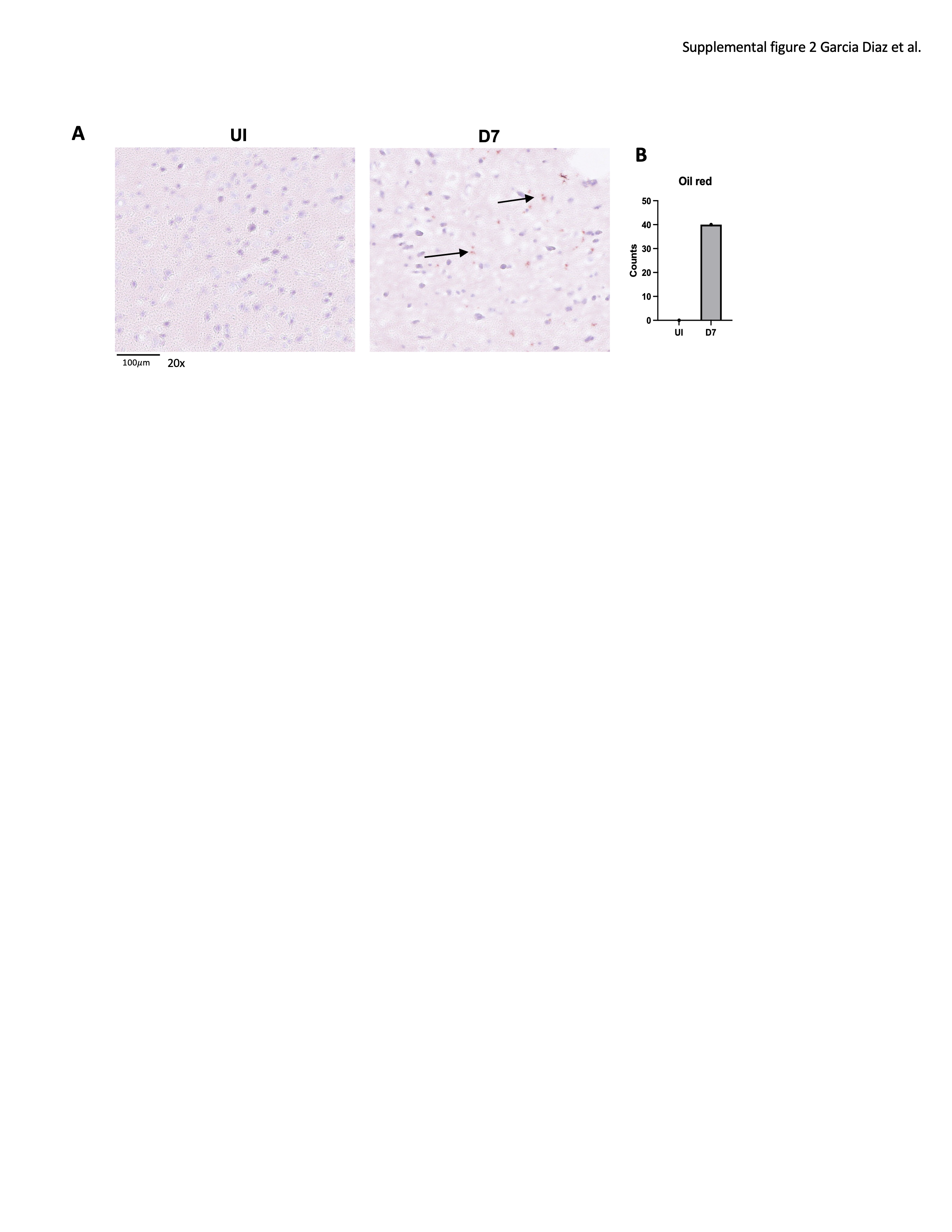

Supplement: Supplementary Figure 2 — Lipid detection observed at 7dpi. (A) Oil red staining of brain tissue with quantification (B). Data represents the mean ± SD ∗p < 0.0332. [file Image2.tiff]

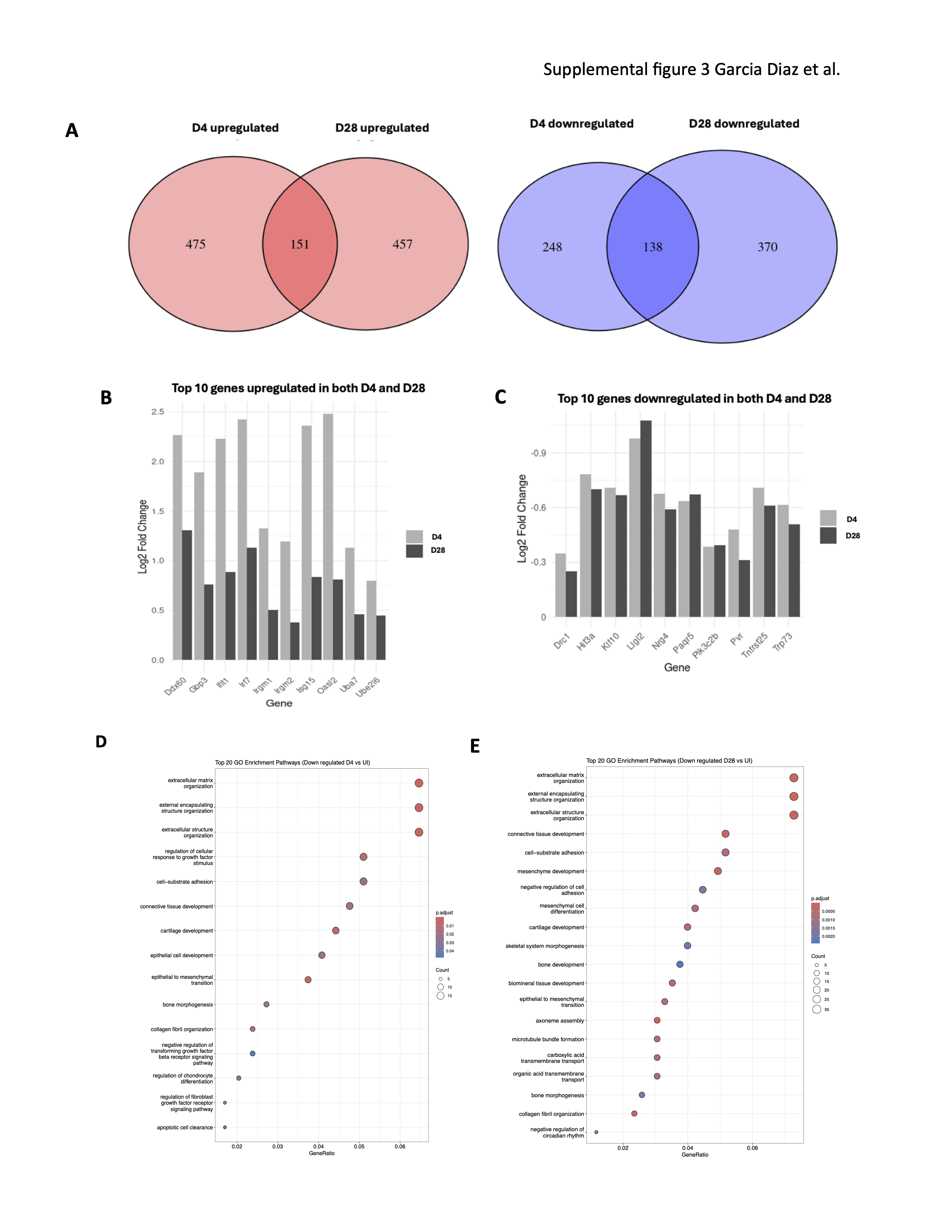

Supplement: Supplementary Figure 3 — Bulk sequencing reveals diverse transcriptomic profile with chronic effects. (A): Left Venn diagram depicts the counts of upregulated genes in D4 and D28 compared to UI that have a p value < 0.05 and a log2foldchange >0. Right Venn diagram shows downregulated genes in D4 and D28 compared to UI that have a p value < 0.05 and a log2foldchange < 0. (B, C) Top 10 genes found to be upregulated/downregulated in both D4 and D28 groups, with a p value < 0.05. (n=3). (D) Gene expression differences comparing D4 to UI visualizing the top most downregulated genes in the D4 group. (E) Gene expression differences comparing D28 to UI visualizing the top most downregulated genes in the D28 group. [file Image3.tiff]

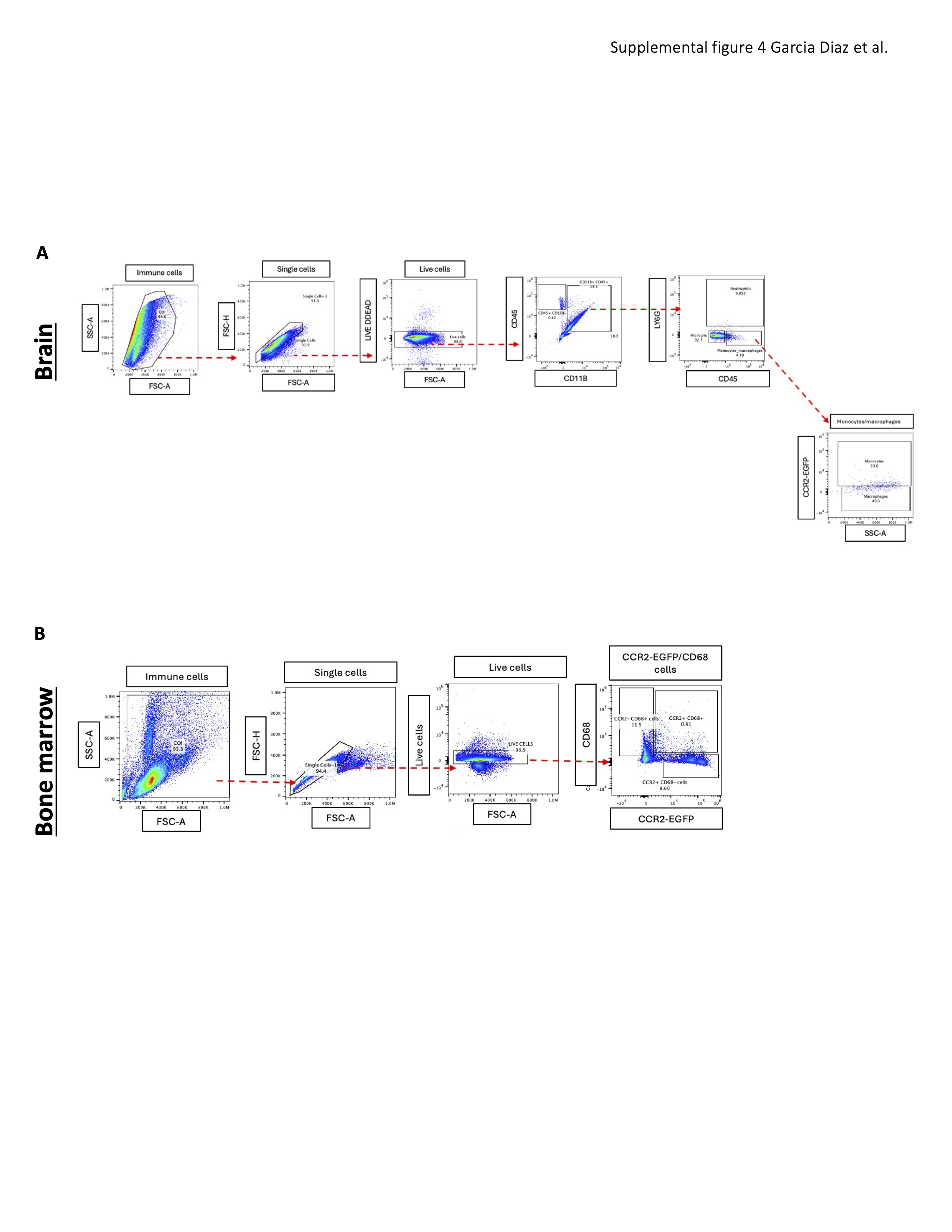

Supplement: Supplementary Figure 4 — Gating strategy for CCR2-creER-R26R EGFP (Ai6) mice for brain (A) and bone marrow (B). [file Image4.tiff]
